# A Cross-Sectional Study of Anesthesia Safety in Wad Medani, Sudan: A Pre-war Status Indicating a Post-war Crisis

**DOI:** 10.7759/cureus.56725

**Published:** 2024-03-22

**Authors:** Alaa I Mohamed, Mohammed S Bashir, Sami M Taha, Yassir M Hassan, Raid M AL Zhranei, Ahmad A Obaid, Abdulrahman M Albarakati

**Affiliations:** 1 Department of Anesthesia Technology, College of Applied Medical Sciences, King Saud Bin Abdulaziz University for Health Sciences, Jeddah, SAU; 2 Research Office, King Abdullah International Medical Research Center, Jeddah, SAU; 3 Department of Urology, University of Gezira, Wad Medani, SDN; 4 Department of Urology, Gezira Hospital for Renal and Urological Surgeries, Wad Medani, SDN; 5 Department of Obstetrics and Gynaecology, University of Gezira, Wad Medani, SDN; 6 Obstetrics and Gynecology, Wad Medani Maternity Hospital, Wad Medani, SDN; 7 Department of Respiratory Therapy, College of Applied Medical Sciences, King Saud Bin Abdulaziz University for Health Sciences, Jeddah, SAU

**Keywords:** low-income countries, war in sudan, health care system, health workforce, safe anesthesia practice

## Abstract

Background: As the surgical burden grows, increasing patient safety during anesthesia and surgery becomes a major global public health priority. Anesthesia can be safely administered in higher-income countries, yet it is more challenging in third-world countries. This study focuses on Sudan, a third-world country, and its unmet anesthetic needs before the current war and how these needs might compromise the post-war status.

Aim: The aim of this study is to compare Sudan's outstanding anesthesia requirements to the World Health Organization's safe anesthesia practice standards in terms of workforce, medications, equipment, and anesthesia conduct.

Methods: This study was carried out in four hospitals (Wad Medani Teaching Hospital, Wad Medani Maternity Hospital, Gezira Centre for Renal and Urological Surgeries, and the National Centre for Pediatric Surgeries) in Wad Medani, two of which were referral and two were state-run. Each hospital from every category was identified using a convenience sampling technique. The World Health Organization-World Federation of Societies of Anesthesiologists International Standard and earlier regional African publications were used to determine the minimum predicted safe anesthesia needs.

Results: The results of our study demonstrate that overall, the hospitals surveyed fulfilled the minimum standards set by the World Health Organization and the World Federation of Societies of Anesthesiologists (WHO-WFSA) for safe anesthesia practice by 73% with no significant difference in the safety of anesthesia practice between state and referral hospitals.

Conclusions: The state of safe anesthesia care in Wad Medani hospitals surveyed fell well short of the expected minimal criteria due to important requirements such as patient monitoring indicators, the inaccessibility of life-saving facilities such as defibrillators, and difficult intubation instruments. More importantly, the conduct of anesthesia was far below the standard.

## Introduction

The World Health Organization (WHO) recognizes that five billion people lack access to safe and affordable surgical and anesthesia services. With 313 million confirmed surgical procedures performed worldwide each year, a percentage of 6% takes place in low- and lower-middle-income countries (LMICs). Even though this percentage is a point of fact, it is considered an underestimate of the actual deficit. This major unmet need is greatest in Africa and South Asia. An estimated 143 million additional surgical procedures should be performed annually to achieve the third goal of the Sustainable Development Goals (SDG3) of saving lives and preventing disabilities by 2030. Improving access to timely, safe, affordable, and high-quality anesthetic and surgical care is critical to reducing these numbers and achieving the SDGs [1].

When comparing high-income countries (HICs) with African countries, there are significant differences in professional training, employment, and retention, as well as differences in population demands and poor working conditions at different levels of the health system [2]. The inadequate distribution of anesthesia providers between HICs and LMICs exacerbates the health workforce dilemma. The presence of shortages and disparities in LMICs, where competent anesthesiologists practice predominantly in metropolitan areas, highlights the same concern. On this basis, enhancing patient safety during anesthesia care and surgical procedures becomes a significant global public health concern [1,2]. For the first time, the World Health Assembly endorsed the expansion of emergency and necessary surgical and anesthetic care as part of universal health care in 2015, along with emphasizing the surgical burden [3].

The safety of anesthesia varies by country, primarily due to economic constraints. Anesthesia is considered extremely safe in HICs but falls behind in LMICs [4-7]. Bashford's study in Ethiopia found that most study participants were unable to safely perform general anesthesia (39%), spinal blocks (50%), pediatric anesthesia (63%), or obstetric (89%) anesthesia [8]. With enhancing anesthetic safety as a main goal, Merry et al. suggested that defining the scope of practices along with developing professional standards, and guidelines, and adopting practice procedures should be developed globally, particularly in LMICs [9].

Current standards of safe anesthesia practice guidelines estimate that they prevent approximately 43% of adverse events in the operating room [10]. The application of these standards in the HICs cannot be denied, although in developing countries it is doubtful due to the multiple burdens that require regular evaluation and reporting [7,11,12]. This makes it more difficult for residents of rural areas to receive quality health care in a timely manner, leading to delays and thereby increasing the number of illnesses, injuries, and impairments that are already threatening the health indicators of these countries [1,6].

In Sudan, until 1998, there were only two institutes that trained anesthesia specialists: the Sudanese Medical Specialization Board for the specialists training and licensing of doctors and the School of Anesthesia Assistants or Technicians [13]. The absence of the former, coupled with the poor outcomes of anesthesia care provided by the latter, prompted the establishment and introduction of Bachelor of Sciences (BSc) programs in anesthesia sciences by the University of Gezira then adopted by other public and private universities [14]. A number of approximately 1200 candidates were graduated from these programs. The majority of the graduates practice in Sudanese hospitals with a variety of tasks and without a defined scope of practice.

This study aimed to compare Sudan's unmet anesthesia needs with the WHO guidelines for safe anesthesia practice with regard to the workforce, medicines, equipment, and the conduct of anesthesia. Although the data for this project was collected before April 2023, it is expected that this analysis will provide the Sudanese Ministries of Health, donors, researchers, organizations, and stakeholders with essential information about the state of anesthesia safety before the war. The results of this analysis will serve as an indicator of measures to implement safe anesthesia practices in the post-war period.

## Materials and methods

This work was part of a larger cross-sectional clinical audit that took place in Wad Medani, Gezira State, Central Region of Sudan, from November 2, 2022, to February 31, 2023. The proposal for this study was revised and approved by the ethics committee of the University of Gezira (UofG) with IRB number 542/2022. The study region was basically selected among Sudan's 18 states as the data collection site since it was Sudan's second-highest-populated city after Khartoum and would represent the degree of anesthesia practice in other major urban areas. Note that prior research studies on anesthesia in Sudan were conducted in Khartoum [13,14]. The study was conducted at four government health institutions that support the mission of global surgery, following previous similar studies that were conducted in the region. Both state hospitals and referral centers were selected: Wad Medani Teaching Hospital, Wad Medani Maternity Hospital, Gezira Centre for Renal and Urological Surgeries, and the National Centre for Pediatric Surgeries. Based on their geographical location and the specialties they provided, these hospitals used to serve over 5.8 million residents of Gezira State, as well as residents from adjacent northern, eastern, and southern states. These centers were preidentified based on a mock survey of Wad Medani hospitals that tested the availability of the WHO minimum standards of safe anesthesia (specifically equipment and medication standards). The questionnaire was designed using the International Standard for the Safe Practice of Anesthesia of the World Health Organization and the World Federation of Societies of Anesthesiologists (WHO-WFSA), along with a previous questionnaire adopted from the WHO-Sample List of Essential Medicines of 2019 and validated by the author, Fassil Mihretu, in Ethiopia [2], as shown in Table 1.

**Table 1:** Minimum safe anesthetic parameters that must be met in each category. Adopted from the WHO-WFSA International Standard for the safe practice of anesthesia and Fassil Mihretu questionnaire. WHO-WFSA: World Health Organization and the World Federation of Societies of Anesthesiologists

Professional aspect	Facilities and equipment	Medications and intravenous fluids	Patient monitoring	Conduct of anesthesia
-Bachelor of Science (BSc) anesthesia professional	“Adequate lighting, tilting operating table, supply of oxygen, Oropharyngeal airways, different size facemasks” Laryngoscopes Endotracheal tubes, intubation aids, suction device, “self-inflating bags, equipment for intravenous (IV) infusions and injection, equipment for spinal anesthesia, sterile gloves, defibrillator, Stethoscope, Pulse oximetry Capnography”, non-invasive blood pressure monitors, and Electrocardiogram	Atropine, Bupivacaine/ atracurium, Ketamine Propofol, Ephedrine Midazolam, Opioids Local anesthetics, Isoflurane, Halothane Nitrous oxide, Sevoflurane, Oxygen Epinephrine	“Clinical observation, using audible signals and alarms, continuous use of pulse oximetry, intermittent” noninvasive blood pressure monitoring. (NIBP), and carbon dioxide detector for patients undergoing intubation	“Preoperative anesthesia assessment and consent, transfer of care and delegation of care, post-anesthesia care unit (PACU), record keeping, WHO safe surgery checklist application, continuous presence of a supervising” anesthesiologist, and pain management

Outcome measurements

The questionnaire included questions regarding professional aspects, facilities and equipment, medications and intravenous fluids, patient monitoring, and the provision of anesthesia, all written in English and not translated into any other language. This availability should be within the facility without placing an undue financial burden on the patient or their family. The questions had only two possible answers: available (yes or 1) and not available (no or 2). Anesthesia practice would be considered safe if the WHO-WFSA minimum requirements for safe anesthesia were completely met, meaning the standard was always available (yes). The questionnaire was distributed to the anesthesia head departments or the medical director at each of the four study sites, depending on which was available at the time of data collection. To acquire our results, we compared the data points collected from the questionnaire to the WHO-WFSA International Minimum Standard for Safe Anesthesia Practice (Table 1). The minimum standard criteria were broken down into five categories: professional elements, facilities and equipment, medications and intravenous fluids, patient monitoring, and anesthesia.

Data processing and analysis

Data were transferred to Microsoft Excel and analyzed using IBM SPSS Statistics for Windows, Version 27 (Released 2020; IBM Corp., Armonk, New York, United States) after ensuring completeness, accuracy, and clarity. The Mann-Whitney test yielded a p-value of <0.05 with a 95% confidence interval. A U-test was applied to examine the safety of anesthesia in referral and state hospitals. Finally, the results were described using a table and a graph.

## Results

Three of the distributed questionnaires were reported by the head of anesthesia, while the pediatric center questionnaire was reported by the center's medical director. In the four hospitals under study, 100 anesthesia providers were documented (Table 2).

**Table 2:** Number and description of anesthesia providers in hospitals under study ^a^An Anesthesia provider: Any healthcare worker who provides anesthesia care, irrespective of professional background or moderate or deep training. ^b ^The description was taken from WHO-WFSA International Standards and the Sudan Ministry of Health [15-18].

^a^Anesthesia provider	^b^Description of anesthesia provider	Total numbers
Anesthesiologist	A graduate of a medical school who is a medical doctor and completed a nationally or internationally recognized specialist anesthesia training program	7
BSc anesthesia technologist	A graduate of a health science college/institute who has completed a nationally recognized BSc anesthesia training program	78
Diploma technician	A graduate of a health science college/institute who has completed a nationally or regionally recognized advanced diploma anesthesia training program	15

Only seven anesthesiologists were identified among these practitioners, with five practicing at Wad Medani Maternity Hospital and none at the pediatric center. The hospitals under study reported 78 BSc anesthesia technologists and 15 Diploma anesthesia technicians. Anesthesiologists were exclusively accessible for elective treatments. Anesthesia for emergency procedures was always provided by at least two technologists and one technician (Table 3).

**Table 3:** Availability of anesthesia providers per day

Anesthesia provider	Recruited				Always Available for Emergency procedures			
	Wad Medani Teaching Hospital	Wad Medani Maternity Hospital	Gezira Center for Urology and Renal Disease	National Center for Pediatric Surgeries	Wad Medani Teaching Hospital	Wad Medani Maternity Hospital	Gezira Center for Urology and Renal Disease	National Center for Pediatric Surgeries
Anesthesiologists	1	5	1	0	0	0	0	0
BSc anesthesia technologists	22	25	15	16	2	2	2	2
Diploma technicians	3	10	0	2	1	1	0	0
Total	26	40	16	18	3	3	2	2

Standards for medications and intravenous fluids

Obtained from the WHO highly recommended drugs of 2021 updated list that should be present in any hospital-based hospital, normal saline, Ringer lactate, and paracetamol/acetaminophen availability were reported in neither hospital (0%), while non-steroidal anti-inflammatory drugs (NSAIDs) and benzodiazepines were reported to be more available. Other medicines, including anesthesia medications, were reported to always be available without any patient hardship (Table 4).

**Table 4:** Availability of medications and intravenous fluids derived from the WHO model list of essential medications in anesthesia NSAIDs: Non-steroidal anti-inflammatory drugs

Medications and intravenous fluids	Available in >75%	Available in 50%	Available in 25%	Not available 0%
Atropine	✔️			
Bupivacaine/Atracurium	✔️			
Ketamine	✔️			
Propofol	✔️			
Ephedrine/ Epinephrine	✔️			
Opioids	✔️			
Isoflurane / Halothane	✔️			
Halothane	✔️			
Nitrous oxide	✔️			
Oxygen	✔️			
Local anesthetics		✔️		
Midazolam/ Diazepam			✔️	
NSAIDs			✔️	
Paracetamol PO				✔️
Normal saline/Ringer’s Lactate				✔️

Facilities and equipment

The availability of items in this standard was almost fairly distributed among the surveyed centers. Defibrillators, difficult intubation aids, and capnography were reported to be available in neither hospital (0%). Electrocardiography was reported only in 25% of the centers as presented in (Table 5).

**Table 5:** Access to facility and equipment NIBP: Non-invasive blood pressure

Facility and Equipment	Available in >75%	Available in 50%	Available in 25%	Not available 0%
Adequate lighting	✔️			
Tilting operating table	✔️			
Oropharyngeal airways	✔️			
Supply of oxygen	✔️			
Anesthesia masks	✔️			
Laryngoscope	✔️			
Endotracheal tubes	✔️			
IV infusions and injection equipment	✔️			
Suction device	✔️			
Self-inflating bags	✔️			
Stethoscope	✔️			
NIBP monitor	✔️			
Pulse oximetry	✔️			
Equipment for spinal anesthesia		✔️		
Sterile gloves			✔️	
Electrocardiogram			✔️	
Defibrillator				✔️
Difficult Intubation aids				✔️
Capnography				✔️

Standards for monitoring

The surveyed hospitals have satisfied 80% of the minimum requirements for safe anesthesia in the category of patient monitoring. Patients were monitored equally in the surveyed hospitals, with a complete absence of capnography monitoring in 100% of the hospitals (Table 6).

**Table 6:** Perioperative patient monitoring NIBP: Non-invasive blood pressure

Perioperative patient monitoring	Almost Always >75%	Often available 50%	Rarely available 25%	Not available 0%
Clinical observation	✔️			
Audible signals and alarms	✔️			
Continuous use of pulse oximetry	✔️			
Noninvasive Intermittent NIBP monitoring	✔️			
Capnography during intubation				✔️

Conduct of anesthesia

**Figure 1:**
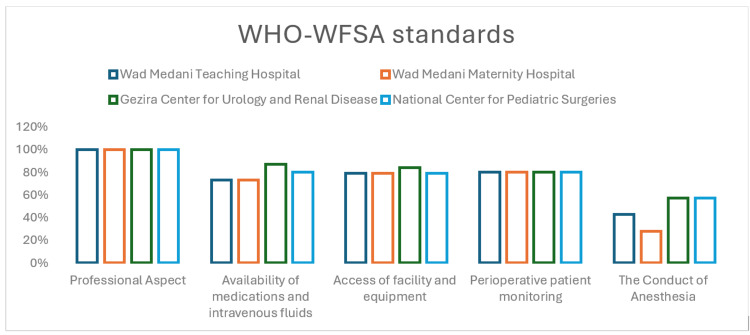
The proportion of WHO-WFSA minimum expected standards for safe anesthesia that were met on each heading in Wad Medani, Sudan WHO-WFSA: World Health Organization and the World Federation of Societies of Anesthesiologists

On average, 49.75% of the assessed hospitals met the minimal required conditions for safe anesthesia practice (Figure 1).

Pre-operative assessments were conducted in all centers under study to invariable degrees, irrespective of the background of the anesthesia provider. Neither the WHO Surgical Safety Checklist nor a modified version were implemented in any hospital. Despite the availability of anesthesiologists in some centers, the practice of anesthesiologist supervision during emergency cases was not reported in any hospital. Also, anesthesia sheet documentation, as well as post-anesthesia requests, have been observed to be evident in one hospital only (Table 7). 

**Table 7:** The Conduct of Anesthesia PACU: Post-anesthesia care unit

Conduct of Anesthesia	Available in >75%	Available in 50%	Available in 25%	Not available 0%
Pre-operative patient evaluation	✔️			
Anesthesiologist supervision / elective surgeries		✔️		
Post-operative care and pain management		✔️		
Documentation			✔️	
Transfer of patients to the PACU with detailed transfer of care			✔️	
Anesthesiologist supervision/emergency surgeries				✔️
Use of the WHO Surgical Safety Checklist				✔️

The Mann-Whitney U test was used to compare the application of the WHO-WFSA standards for safe anesthesia among centers and to test whether there was a significant substantial correlation with the hospital level. Safe anesthesia practice was found to be not correlated to the hospital level (p > 0.01) (Table 8).

**Table 8:** Comparison between state and referral hospitals in meeting the minimum safe anesthesia requirements

Hospital category	Meets minimum safe anesthesia standards (%)	Doesn’t meet minimum safe anesthesia standards (%)	p-value
State Hospital	74	26	>0.01
Referral Hospitals	80.4	19.6	

## Discussion

The results of our study demonstrate that overall, the hospitals surveyed fulfilled the expected minimum standards set by the WHO-WFSA for safe anesthesia practice by 74%. Adequate quantities of basic anesthetics, analgesics, resuscitation agents, other adjuvants, intravenous fluids, equipment, and facilities should always be provided to every patient, without any financial burden to the patient [11]. Yet, like the majority of low-income countries, including Sudan, this cannot be afforded, especially after the current conflict [4-8]. Our results showed that the level of safe anesthesia practice in Wad Medani was consistent with other LMICs.

Unlike previous studies, this study used “yes” or “no” options to describe the absolute availability or unavailability of items within each data collection criterion. It is reasonable to expect that after the war, the percentage of hospitals meeting the minimum criterion will decline significantly due to continued violations of facilities and workforce as collateral or intended damage of the warfires. The researchers believed that this method better reflected the state of practice in Sudan than other surveys that used almost always, frequently, and rarely options in the assessment tool.

Although not meeting the WHO standard, the BSc anesthesia technologists were pre-identified for the minimum standard of care in the professional component, not anesthesiologists, similar to a previous study conducted in Ethiopia. All evaluated hospitals always have at least 10% of anesthesia technologists with bachelor's degrees available to cover the majority of anesthesia care. The statistics of anesthesia providers in Sudan have increased, and during the data collection, it was proportionate to the number of anesthesia practitioners in all regions of the country in 2006 [13]. In the past, the lack of qualified anesthesiologists in Sudan and other LMICs resulted in anesthesia being performed by untrained medical professionals, with subsequent patient referrals out of state to other hospitals [13,14]. Anesthesiologists with extensive training and specialty experience were distributed only within major cities and large referral hospitals. Apart from the extreme shortage of anesthesiologists, there had been a considerable number of BSc anesthesiologists who, unfortunately, were working without a defined scope of practice.

Previous studies that included regional LMICs acknowledged shortages of equipment, facilities, essential medications, and monitoring devices, along with a Sudanese study by Ahmed et al. in Khartoum [6,7,13-19]. Our findings were comparable to those of other underdeveloped countries and in Khartoum in terms of necessary medicines and equipment, with little significant improvement. Despite using the updated 2021 WHO list of safe anesthesia practices, as opposed to previous studies that used 2019 and earlier versions, ketamine and atropine, for instance, were available in all hospitals examined. This result is much higher than the results of the Bashford and Fassil study in Ethiopia and Hodges et al. in Uganda [8,20-23]. The availability of medications in the Bashford study by Hodges et al. appears to be lower than in this survey, which might be related to the inclusion of primary hospitals by Bashford, which would be predicted in our results if we included these types of healthcare facilities in our study. Even though intravenous fluids were still provided by surgical patients in all centers under study, this is a significant indicator of noncompliance with the WHO-WFSA and UHC mandates, which require healthcare facilities to provide these medications without applying any financial burdens on patients.

In a previous study that included 22 LMICs and which adopted the WHO Assessment tool for facility and equipment version 2009, a percentage of 45.2% of the surveyed facilities had an uninterrupted supply of oxygen via either oxygen concentrators or cylinders, which is much lower than the results of our survey [17-21]. Historically, many LMICs, including Sudan, have had no or limited access to pulse oximetry [24-26]. The results of our study showed that 100% of the examined facilities always had pulse oximetry, demonstrating an improvement in anesthesia practice in several areas. However, devices particularly critical to saving patient lives, such as defibrillators and difficult airway management, were in great deficit and required significant investment. Confirming that cardiac arrest and difficult airway cascades are the leading causes of anesthesia-related mortality [27]. This should definitely be augmented for the post-war period.

The lack of equipment and infrastructure negatively impacts patient monitoring, which is one of the biggest hurdles to accessing safer anesthesia care. Evidence suggests that the use of clinical observation, physical examination, and key monitoring devices in patient monitoring improves perioperative patient outcomes and enhances patient safety [20,28,29]. Clinical observation includes monitoring pulse rate and quality and visualizing tissue oxygenation and perfusion. Other measures of clinical observation include observing breathing bag movement, listening to breath sounds and heart sounds, and determining pain levels and the need for analgesia. It is well noticed that in 100% of the hospitals surveyed, patients were always monitored through clinical observation, continuous use of pulse oximetry, and NIBP monitoring. In contrast, carbon dioxide detectors have been found to be unavailable for patients undergoing intubation.

Unlike previous similar studies, the overall quality of anesthesia in the four centers was rather poor compared to other standards, including facilities and medications. The results demonstrated an extreme delay in the implementation of certain standards, particularly the application of the WHO Surgical Safety Checklist or its modified forms along with documentation and the availability of anesthesiologists during emergency procedures. This delay negatively affects the overall standard of anesthesia delivery. The application of the WHO Surgical Safety Checklist is known to reduce anesthesia- and surgery-related mortality and morbidity [30-32]. Similar previous surveys by Bashford et al. [33] and Eshete et al. [34]. in Ethiopia and Uganda, respectively, found that the use of the WHO checklist in their standard data was also low. Inadequate post-operative pain care was observed in the evaluated hospitals, as demonstrated by a comparable study in Uganda and Ethiopia [35].

Although higher-level (referral) hospitals might clinically perform anesthesia more safely than district hospitals in terms of basic requirements, there was no statistical significance. Higher-level hospitals are expected to meet certain standards. One of the limitations of the study was that it did not examine whether any of the hospitals met higher WHO requirements (suggested and recommended).

## Conclusions

The safety of anesthesia care in hospitals in Wad Medani, Sudan, was far from expected standards before the current war due to key requirements such as patient monitoring indicators, life-saving facilities, and inadequate supervision. The recent war has negatively impacted anesthesia care in Sudan, particularly in Khartoum, Wad Medani, and Darfur among other regions. To meet minimum standards, heavy investment in medical education and training is essential, along with investments in essential equipment, medications, and facilities.

The Sudanese health authorities, in collaboration with relevant universities and health organizations, should promote anesthesia technology careers by defining and publishing the scope of practice and providing fair legal and financial commitments. Recruitment of highly trained MSc technologists can cover to a great extent the shortage of anesthesiologists especially in rural and marginal areas. Further research is needed to fill information gaps regarding anesthesia staff availability, oxygen concentrators, and the supply of medicines and equipment. The WHO defines highly recommended standards as the functional equivalent of mandatory standards, and the Ministry of Health should ensure these standards are met urgently.
